# Comparison of three real-time polymerase chain reaction protocols for the diagnosis of imported schistosomiasis in a non-endemic setting

**DOI:** 10.1186/s13071-025-07203-1

**Published:** 2025-12-29

**Authors:** Patricia Martínez-Vallejo, Aroa Silgado, Alejandro Mediavilla, Carles Rubio Maturana, Francesc Zarzuela, Marc Muixí, Lidia Goterris, Esther Rodríguez, Sara Vázquez, Fernando Salvador, Inés Oliveira-Souto, Israel Molina, Núria Serre-Delcor, Javier Sotillo, Elena Sulleiro

**Affiliations:** 1 Microbiology Department, Vall d’Hebron University Hospital, Vall d’Hebron Research Institute (VHIR), PROSICS Barcelona, Barcelona, Spain; 2 Department of Microbiology and Genetics, Universitat Autònoma de Barcelona (UAB), Barcelona, Spain; 3 Centro de Investigación Biomédica en Red de Enfermedades Infecciosas (CIBERINFEC), Instituto de Salud Carlos III, Madrid, Spain; 4 International Health Unit Vall D’Hebron-Drassanes, Infectious Diseases Department, University Hospital Vall d’Hebron, PROSICS Barcelona, Barcelona, Spain; 5 Parasitology Reference and Research Laboratory, Centro Nacional de Microbiología, Instituto Salud Carlos III, Madrid, Spain

**Keywords:** Real-time polymerase chain reaction, *Schistosoma*, Imported schistosomiasis, Dra1, Sm1-7

## Abstract

**Background:**

Schistosomiasis is a neglected tropical disease that mostly affects inhabitants of sub-Saharan Africa. With rising global migration, imported cases of schistosomiasis are increasingly being reported in non-endemic countries, where diagnosis is hindered by low parasite burdens and multiple *Schistosoma* species. Microscopy remains the gold standard, despite its limitations, whereas molecular techniques offer greater sensitivity. The aim of this study was to assess the performance of real-time polymerase chain reaction (PCR) protocols for the detection, at an international health centre in Barcelona, of imported cases of urogenital and intestinal schistosomiasis.

**Methods:**

This cross-sectional study included 75 adults from sub-Saharan Africa attending the Drassanes-Vall d’Hebron International Health Unit, Barcelona, between May 2023 and February 2024. Paired urine and stool samples were collected. Microscopy was performed on all samples. Urine was analysed by real-time PCR using the Dra1 target sequence. Stool was tested by three protocols targeting, respectively, Dra1, Sm1-7, and 28S rRNA. *Schistosoma* infection was confirmed by microscopic identification of eggs and/or parasite DNA detection by real-time PCR.

**Results:**

Schistosomiasis was confirmed in 12/75 patients (16%). Urogenital schistosomiasis was diagnosed in 3/75 cases; the performance values of real-time PCR in urine samples were not assessed. In stool, the pan-*Schistosoma* real-time PCR showed 55.6% sensitivity and 98.5% specificity, with a moderate agreement (*κ* = 0.631) with microscopy. The Sm1-7 assay fully matched microscopy for *Schistosoma mansoni* detection, and reached 100% sensitivity and specificity. A novel contribution of this study is the application of a real-time PCR assay targeting the Dra1 repetitive sequence in stool samples for the detection of *Schistosoma intercalatum/Schistosoma guineensis*. All of the microscopy-positive cases were real-time PCR positive, and one additional infection was detected by real-time PCR, which meant that 100% sensitivity and 98.6% specificity were achieved with this technique.

**Conclusions:**

Our findings underscore the need for accurate diagnostic tools for cases of imported schistosomiasis in non-endemic settings. Microscopy remains the reference standard, while the pan-*Schistosoma* real-time PCR showed limited sensitivity for stool samples. In contrast, the Sm1-7 and Dra1 assays demonstrated higher sensitivity and strong concordance with microscopy, with Dra1 also proving useful for the detection of *S. intercalatum*/*S. guineensis* in stool.

**Graphical abstract:**

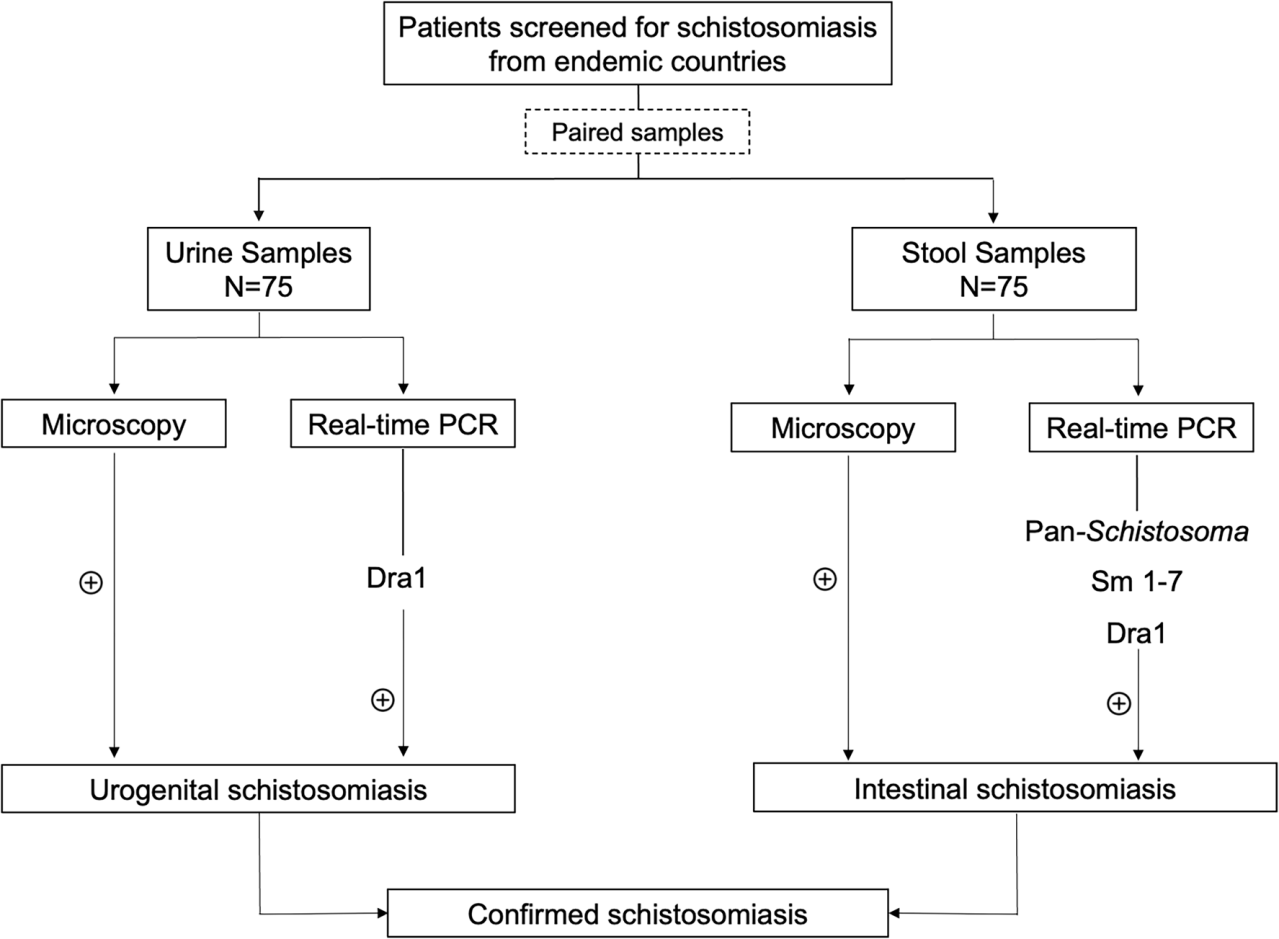

**Supplementary Information:**

The online version contains supplementary material available at 10.1186/s13071-025-07203-1.

## Background

Schistosomiasis is a neglected tropical disease, with more than 250 million people infected worldwide, the vast majority of whom live in sub-Saharan Africa [1, 2]. Human schistosomiasis can be caused by *Schistosoma haematobium*, *Schistosoma mansoni*, *Schistosoma japonicum*, *Schistosoma mekongi*, *Schistosoma intercalatum*, and *Schistosoma guineensis*, with the first three species having the greatest clinical and socioeconomic importance. In non-endemic settings, such as Europe, schistosomiasis is primarily detected among migrants and travellers, mainly from Africa. However, diagnosis is frequently missed or delayed due to low clinical suspicion and the diagnostic challenges associated with this neglected tropical disease [3, 4].

In sub-Saharan Africa, schistosomiasis prevalence varies considerably across geographic regions, with marked heterogeneity observed between countries, districts, and villages. The main burden of disease is usually attributed to *S. mansoni* and *S. haematobium*, while infections with *S. intercalatum and S. guineensis* contribute to a lesser extent [5]. The clinical manifestations and long-term morbidity associated with schistosomiasis are primarily due to the host's immune response to the parasite’s eggs. Chronic infection results in persistent inflammation and tissue damage, often leading to complications years after the initial infection [3].

*Schistosoma haematobium* adult worms reside in the venous plexus of the bladder, and cause urogenital schistosomiasis (UGS) in humans [6]. In addition, *S. haematobium* is a recognised carcinogen and represents the second leading cause of bladder cancer worldwide and genital disorders [7]. Chronic infection is also associated with infertility and an increased susceptibility to other infections, including human immunodeficiency virus and human papillomavirus [8, 9]. *Schistosoma mansoni*, *S. intercalatum*, and *S. guineensis* reside in the mesenteric vein, causing intestinal schistosomiasis, and clinical manifestations may include blood in the stool, constipation, and diarrhoea. Chronic inflammation can lead to bowel wall ulceration, hyperplasia, and the development of intestinal polyps [10–12].

The current gold standard for the parasitological diagnosis of schistosomiasis is based on the detection of *Schistosoma* eggs by microscopic examination. Depending on the species, the eggs are identified in stool samples for intestinal schistosomiasis or in urine for UGS. The most commonly used techniques include the Kato-Katz method, which allows quantification of eggs per gram of faeces, and various concentration techniques. For the detection of *S. haematobium* eggs, urine filtration, sedimentation, or centrifugation methods are commonly employed [1]. These parasitological methods are useful in high-prevalence settings; however, they have limited sensitivity for the detection of low-intensity infections, such as those typically found in non-endemic areas, thereby increasing the risk of misdiagnosis [13, 14].

Accurate diagnosis enables timely treatment and prevents severe long-term complications. Furthermore, it is essential for disease control and eventual elimination, and enables the identification of new cases or transmission foci and the monitoring of the effectiveness of control strategies [15]. Molecular assays, such as the polymerase chain reaction (PCR), are highly sensitive and specific tools for diagnosing schistosomiasis. PCR-based methods can detect the presence of parasite DNA even in the absence of visible eggs [16, 17]. This is particularly important in prepatent infections, during the acute phase of the disease, when worms have not yet produced large numbers of eggs, as well as in active and chronic infections with low egg loads, which are frequently observed in travellers and migrants in non-endemic countries [12, 18, 19].

Several *Schistosoma* DNA targets have been described [20, 21]. Some of the most commonly used PCR targets include the Dra1 sequence of the *Schistosoma haematobium* species clade, which includes *Schistosoma haematobium*, *Schistosoma intercalatum*, *Schistosoma guineensis* and *Schistosoma bovis*, and the Sm1-7 tandem repeat sequence of the *Schistosoma mansoni* species clade, which includes *S. mansoni* and shows cross-reactivity with *S. bovis* from the *S. haematobium* clade [22]. Additionally, a PCR-based approach targeting a specific region of the 28S large subunit ribosomal RNA gene has also been described [18], enabling genus-specific amplification of all *Schistosoma* species. This method allows for the detection of DNA from five *Schistosoma* species: *S. mansoni*, *S. haematobium*, *S. intercalatum/S. guineensis*, *S. japonicum*, and *S. mekongi* [23]. While all of these techniques demonstrated greater sensitivity than conventional microscopy, the real-time PCR assays targeting Dra1 and Sm1-7 sequences (with detection limits of < 10 fg and 1 fg respectively) exhibited a lower limit of detection compared to the real-time PCR targeting the 28S subunit (0.01 ng). This difference could impact the overall sensitivity of that assay and, consequently, the accuracy of patient diagnosis [24–26].

Three real-time PCR protocols targeting the Dra1 [27], Sm1-7 [18], and 28S rRNA [28] (pan-*Schistosoma*) sequences have been previously described as sensitive and robust molecular diagnostic tools. This study aimed to evaluate their applicability and comparative diagnostic performance for the detection of imported cases of urogenital and intestinal schistosomiasis in a referral centre in Barcelona.

## Methods

### Study design and sample collection

**Fig. 1:**
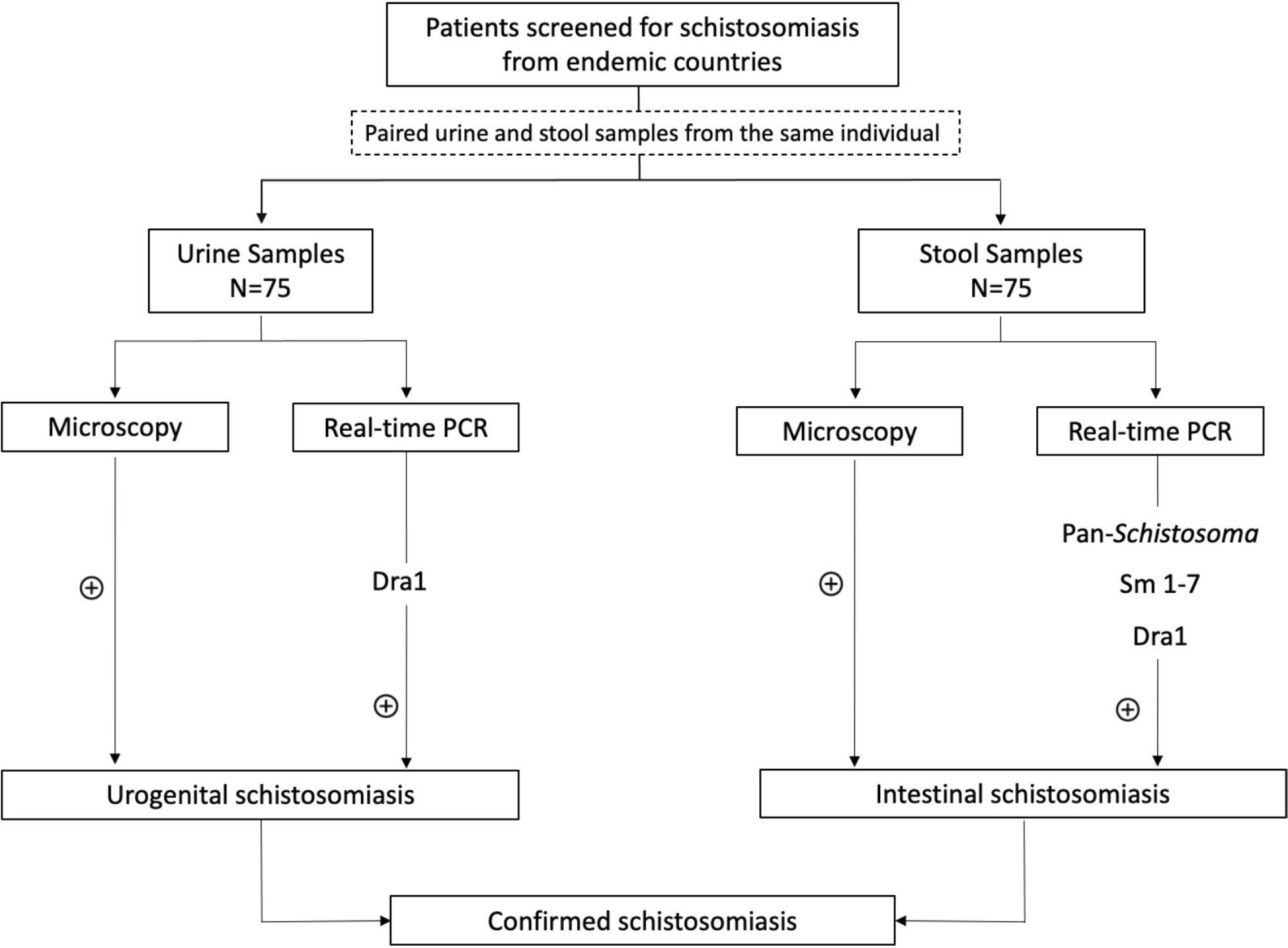
Flow diagram of the study. For real-time polymerase chain reaction (PCR) assays, the targets used in each case are specified

A cross-sectional study was performed at the International Health Centre Drassanes-Vall d’Hebron. Seventy-five adults from sub-Saharan Africa who attended the centre between May 2023 and February 2024 were included in the study. Paired urine and stool samples were collected from symptomatic/asymptomatic patients from countries endemic for schistosomiasis, i.e. mainly migrants and visiting friends and relatives. Both urine and stool samples were analysed by microscopic examination for the visualisation of *Schistosoma* eggs following the protocols of our reference centre [29]. Samples were kept at 4 °C if DNA extraction was performed within a few days, or at − 80 °C for longer-term storage. Additionally, three real-time PCR protocols based on the amplification of Dra1, Sm1-7, and 28S rRNA (pan-*Schistosoma*), respectively, were used for detecting *Schistosoma* DNA. In the absence of an established gold standard, *Schistosoma* infection was defined in our study as confirmed if eggs were visualised by microscopy and/or a positive result was obtained by real-time PCR (Fig. 1). Microscopic examination was performed at the International Health Centre Drassanes-Vall d’Hebron, and real-time PCR protocols were conducted at the Microbiology Department of Hospital Universitario Vall d’Hebron (Barcelona, Spain) and the National Centre for Microbiology-Instituto de Salud Carlos III (Madrid, Spain).

### Diagnostic methods

#### Microscopy examination

UGS was diagnosed by detecting parasite eggs in urine samples. A minimum of 10 mL of urine was collected and processed by sedimentation in accordance with World Health Organization guidelines for the diagnosis of schistosomiasis [1]. Samples were centrifuged at 423 *g* for 5 min, and the resulting sediments were immediately examined under a light microscope. Stool samples were processed following Ritchie’s formalin-ether concentration technique, with some modifications. Approximately 2–3 g of faeces was mixed with 10 mL of 4% formaldehyde and homogenized to form a uniform emulsion. The mixture was strained through double-layer gauze into a Falcon tube, adjusted to 8 mL with formaldehyde, and 2 mL of diethyl ether was added. Samples were centrifuged at 423 *g* for 3 min and the supernatant discarded to allow visualization of the sediment. For both urine and stool samples, *Schistosoma* species identification was based on egg morphology [12].

#### Nucleic acid extraction

DNA extraction from urine samples was performed using the QIAsymphony SP System (Qiagen, Hilden, Germany) at the Microbiology Laboratory of the Hospital Universitario Vall d’Hebron. A total of 1 mL of centrifuged urine was processed and eluted in 110 µL of elution buffer, according to the manufacturer’s instructions.

Aliquots of the stool samples were sent to the Reference and Research Laboratory of Parasitology at the National Centre for Microbiology (Majadahonda, Spain) for DNA extraction and real-time PCR analysis. DNA extraction was carried out on 200 mg of each faecal sample using the QIAamp PowerFecal Pro DNA kit (Qiagen) on the QIAcube System (Qiagen), following the manufacturer’s instructions. The extracted DNA was eluted in 200 μL elution buffer and stored at − 20 °C until use.

#### Real-time PCR

Different approaches based on real-time PCR were employed; the primers and probes used for each real-time PCR protocol are shown in Table 1. In the case of urine samples, to detect *S. haematobium*, a duplex real-time PCR assay targeting the 121 base pair (bp) tandem repeat Dra1 sequence of *S. haematobium* species clade and the human RNase P gene (Taq Man Human Rnase P detection reagent; Applied Biosystems, Foster City, CA) was performed, with Rnase P used as an internal control [27]. Briefly, the final conditions in the PCR mixture were 1× QuantiTec Multiplex PCR kit (Qiagen), 160 nM of both primers for the Dra1 target, 80 nM of the TaqMan Dra1 probe, and 0.8× Rnase P detection reagent. Reactions were performed using 5 µL of eluted DNA in a final volume of 25 µL. Amplifications were carried out on a CFX96 Touch Real-Time PCR Detection System (Bio-Rad, Hercules, CA). The cycling conditions were as follows: denaturation at 95 °C for 10 min, followed by 50 cycles at 95 °C for 15 s and 60 °C for 1 min.

**Table 1:** Primers and probes used in the different real-time polymerase chain reaction (PCR) protocols

Target sequence	Primers and probe	Oligonucleotide sequence (5ʹ-3ʹ)	References
Dra1	Sh-Fw	GATCTCACCTATCAGACGAAAC	[27]
	Sh-Rv	TCACAACGATACGACCAAC	
	Sh-P	FAM-TGTTGGTGGAAGTGCCTGTTTCGCAA-IowaBlack FQ	
Sm1-7	SRA1	CCACGCTCTCGCAAATAATCT	[28]
	SRS2	CAACCGTTCTATGAAAATCGTTGT	
	SRP	FAM-TCCGAAACCACTGGACGGATTTTTATGAT-TAMRA	
28S rRNA Pan-*Schistosoma*	FW	TGGAGTTGAACTGCAAGC	[18]
	RV1	CCATAGCAGACAGGCAGC	
	RV2	GCTCAACAWTAATAGTCAAACCTG	
	Probe	FAM-ACTGACAAGCAGACCCTCACACC-BHQ1	
Hs_18S	FW	GAGCCGCCTGGATACCGC	[40]
	RV	GACGGTATCTGATCGTCTTC	
	Probe	HEX-TCGCTCTGG-ZEN-TCCGTCTTG-3IABkFQ	

For stool samples, three different real-time PCR protocols were performed, with the following objectives: (1) to detect *S. intercalatum* by using the previously described assay targeting the Dra1 sequence of the *S. haematobium* species clade, (2) to detect *S. mansoni* by using a duplex real-time PCR targeting the highly repeated 121-bp Sm1-7 sequence of *S. mansoni* and the human 18S rRNA gene, used as an internal control; and (3) to detect species of the *Schistosoma* genus by using a real-time PCR targeting the 28S rRNA gene of the genus *Schistosoma* (pan-*Schistosoma*). The Sm1-7 assay was performed according to Hamburger et al. [28] by using 5 µL of eluted DNA in a final reaction volume of 20 µL. Thermal cycling conditions were 95 °C for 5 min, followed by 45 cycles of 95 °C for 15 s and 60 °C for 30 s. The pan-*Schistosoma* real-time PCR targeting the 28S rRNA gene was performed according to a published protocol [18]. Each reaction contained 5 μL of eluted DNA in a total volume of 25 μL, comprising 40 nM of each primer (FW, RV1, and RV2), 600 nM of the probe, and 0.1 mg/mL bovine serum albumin. The thermal profile consisted of an initial denaturation step at 95 °C for 2 min, followed by 50 cycles of 95 °C for 15 s, 60 °C for 30 s, and 72 °C for 30 s.

Amplification of DNA isolated from stool was conducted on a Rotor-Gene Q (Qiagen), with all samples tested in duplicate. Each real-time PCR run included a no-template control (nuclease-free water) and a positive control corresponding to the target species. For the Sm1-7 assay, DNA, extracted from the stool of a patient who had been confirmed positive for *S. mansoni* by both microscopy and real-time PCR, was used as the positive control. For the Dra1 and pan-*Schistosoma* assays, DNA extracted from the urine of a patient who had been confirmed positive for *S. haematobium* was used. A sample was considered positive when amplification occurred within 40 cycles (Ct < 40), a threshold adopted from previous optimisation studies and widely used, since late-cycle signals were consistently unspecific. Samples were considered invalid when the internal control was inefficiently amplified.

### Statistical analysis

All data were analysed using the R-UCA package for Windows (version R-UCA-3.3.1.exe; R Development Core Team, Vienna, Austria). Qualitative variables were expressed as absolute frequencies and percentages. Quantitative variables were described as mean and SD, or as medians and interquartile ranges, depending on the distribution. Diagnostic performance parameters were calculated, including sensitivity, specificity, positive predictive value, and negative predictive value. Cohen’s kappa coefficient was used to assess the agreement between each test.

## Results

### Study population and case identification

**Fig. 2:**
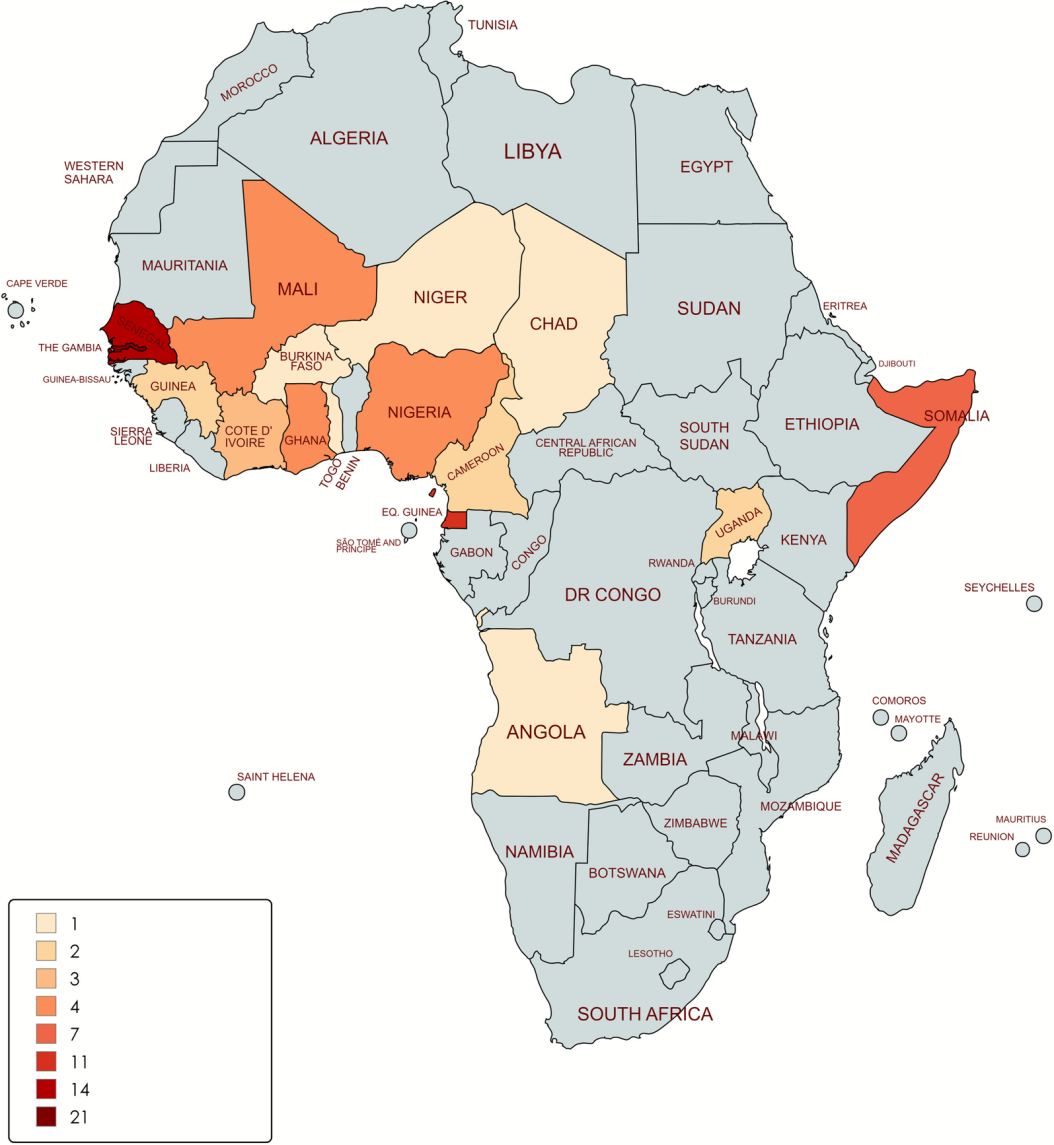
Geographical origin of the 75 patients included in the study and screened for schistosomiasis. The map was generated using MapChart (https://www.mapchart.net/)

Of the 75 study patients, 64/75 (85.33%) were male, with a median age of 23 years (interquartile range 17–34). All of the patients were originally from sub-Saharan Africa, with the majority from Gambia (21/75, 28%), followed by Senegal (14/75, 18.67%) and Equatorial Guinea (11/75, 14.67%) (Fig. 2). *Schistosoma* infection was confirmed in 12/75 (16%), defined as a confirmed case of infection based on a positive result by either microscopy or real-time PCR. Among these, 2/12 (16.67%) cases corresponded to UGS caused by *S. haematobium,* with patients from Senegal and Gambia; 9/12 (75%) cases corresponded to intestinal infections, including 3/12 (25%) of single infections due to *S. mansoni* from Guinea Conakry and Senegal; and 6/12 (50%) were due to *S. intercalatum/S. guineensis*, all of which originated from Equatorial Guinea; and 1/12 (8.33%) case corresponded to a co-infection with *S. haematobium* and *S. mansoni* from Gambia. Representative images from the microscopy examinations, illustrating the classification and differentiation of *Schistosoma* spp. species based on egg morphology, are provided in Supplementary Material Figure S1.

### Urine sample analysis

All 75 urine samples were analysed using microscopy and real-time PCR targeting Dra1, identifying 3/75 (4%) cases of UGS: one diagnosed by both techniques, one by microscopy alone, and one by real-time PCR alone. The mean Ct value for real-time PCR-positive cases was 35.37 (range 34.39–36.34, SD 1.38).

### Stool sample analysis

#### Detection of intestinal schistosomiasis

Analysis of stool samples revealed a total of 10/75 (13.33%) cases of intestinal schistosomiasis. Among these confirmed cases, 4/75 (5.3%) were infected with *S. mansoni*, while 6/75 (8%) were infected with *S. intercalatum/S. guineensis*. Of the 10 positive cases, 9/10 were confirmed by both microscopy and real-time PCR, while in 1/10 case, corresponding to *S. intercalatum/S. guineensis*, detection was by real-time PCR only.

Cases were confirmed by real-time PCR when at least one of the specific targets (pan-*Schistosoma*, Sm1-7, or Dra1) yielded a positive result. Although the Dra1 target is traditionally used for *S. haematobium* detection in urine, in our study, Dra1 was amplified from stool samples containing eggs morphologically compatible with those of *S. intercalatum/S. guineensis*, suggesting that this target may be present in *S. intercalatum/S. guineensis* DNA. The results for the positive samples obtained using each diagnostic technique are detailed in Table 2.

**Table 2:** Diagnostic test results for confirmed cases of intestinal schistosomiasis

Identifier	Microscopy			Real-time PCR			
	*Schistosoma mansoni*	*Schistosoma intercalatum/S. guineensis*	Final result	Pan-*Schistosoma* (Ct)	Sm1-7 (Ct)	Dra1 (Ct)	Final result
13	−	−	−	+ (33.32)	−	+ (17.53)	**+**
46	**+**	−	**+**	+ (21.00)	+ (15.59)	−	**+**
55	**+**	−	**+**	+ (21.70)	+ (12.08)	−	**+**
57	−	**+**	**+**	+ (29.41)	−	+ (16.97)	**+**
58	−	**+**	**+**	+ (34.65)	−	+ (20.20)	**+**
61	**+**	−	**+**	−	+ (28.44)	−	**+**
62	−	**+**	**+**	−	−	+ (23.71)	**+**
68	**+**	−	**+**	+ (27.13)	+ (20.50)	−	**+**
69	−	**+**	**+**	−	−	+ (24.16)	**+**
75	−	**+**	**+**	−	−	+ (25.20)	**+**
No. of positives	4	5	9	6	4	6	10
Ct (mean)				27.87	19.15	21.30	
SD				5.72	7.09	3.56	

*Ct* Cycle threshold

### Diagnostic performance of real-time PCR for stool samples

The diagnostic performance of the three real-time PCR protocols targeting 28S rRNA (pan-*Schistosoma)*, Sm1-7, and Dra1 is summarised in Table 3. Sensitivity, specificity, predictive values, and the kappa coefficient are presented for each assay.

**Table 3:** Diagnostic performance of the three real-time PCR protocols (Pan-*Schistosoma*, Sm1-7, and Dra1), with microscopy as the reference standard

	Microscopy (reference method)			Sn (95% CI)	Sp (95% CI)	PPV (95% CI)	NPV (95% CI)	κ (95% CI)	% Agreement
	Positive	Negative	Total						
Pan-*Schistosoma*									
Positive	5	1	6	55.56 (22.65–84.66)	98.48 (90.73–99.92)	83.33 (36.48–99.12)	94.20 (85.07–98.13)	0.631 (0.336–0.926)	93.33
Negative	4	65	69						
Total	9	66	75						
Sm1-7^a^									
Positive	4	0	4	100 (39.58–97.65)	100 (93.60–99.87)	100 (39.58–97.65)	100 (93.60–99.87)	1	100
Negative	0	71	71						
Total	4	71	75						
Dra1^b^									
Positive	5	1	6	100 (46.29–98.13)	98.57 (91.23–99.93)	83.33 (36.48–99.12)	100 (93.43–99.87)	0.902 (0.71–1)	98.67
Negative	0	69	69						
Total	5	70	75						

*Sn* Sensitivity, *Sp* specificity, *PPV* positive predictive value, *NPV* negative predictive value,* CI* confidence interval

^a^Sm1-7 real-time PCR was evaluated with respect to samples in which *Schistosoma mansoni* was detected by microscopy only

^b^Dra1 real-time PCR was evaluated with respect to samples in which *Schistosoma intercalatum/S. guineensis* was detected by microscopy only

For the Sm1-7 target*,* of the four samples confirmed positive for *S. mansoni*, three were positive for both assays, while one was positive only with the Sm1-7 real-time PCR. The remaining 71 samples were negative for both tests. Among the six samples confirmed positive for *S. intercalatum/S. guineensis*, three were positive for both the Dra1 and pan-*Schistosoma* assays, while the other three were positive only for the Dra1 real-time PCR. A total of 69 samples were negative for both assays.

## Discussion

In recent years, increasing migrant flows to Europe have led to a rise in imported cases of schistosomiasis, and there has also been an increase in imported cases among travellers returning from countries endemic for this disease [30]. In non-endemic regions, schistosomiasis presents numerous clinical and diagnostic challenges for its appropriate management and monitoring. Patients often present with low parasite loads, complicating diagnosis and increasing the risk of severe complications when left untreated [2, 31].

Studies have reported a schistosomiasis prevalence of 12.3% among African migrants who first arrived in Spain between October 2004 and February 2017, with their diagnosis based on the direct visualisation of schistosome eggs [32, 33]. In the present study, *Schistosoma* infection was diagnosed in 16% of patients by using both microscopy and real-time PCR. UGS was diagnosed in only 4% of the patients, while 13.3% were confirmed to have intestinal schistosomiasis. These proportions reflect the selected study population, which included only patients with paired stool and urine samples, and should not be interpreted as representing the prevalence in the general migrant population. The evaluation of real-time PCR performance for the diagnosis of UGS was not considered feasible in this study due to the limited number of positive urine samples. A previous study that was also conducted at the International Health Centre, Drassanes-Vall d’Hebron, reported a 5.3% prevalence of imported UGS between November 2021 and December 2022, based on microscopy and real-time PCR analysis, with the latter showing higher sensitivity (87.5% vs 75%) [34].

Regarding the diagnostic performance of the different real-time PCR protocols for intestinal schistosomiasis, a previous study reported high sensitivity of the real-time PCR targeting the 28S rRNA gene for the detection of *Schistosoma* spp. DNA in stool samples, with an even greater sensitivity than that of microscopy [18]. However, in our study, a higher number of positive cases were detected when using microscopy than the pan-*Schistosoma* real-time PCR, which had a lower observed sensitivity and moderate concordance with the former (κ = 0.63). However, pan-*Schistosoma* real-time PCR showed high specificity and identified one additional positive case that was missed by microscopy that likely reflected a low-intensity infection. Based on these findings, the current pan-*Schistosoma* real-time PCR protocol may not be suitable for large-scale screening purposes [35]. In contrast, the real-time PCR targeting the Sm1-7 sequence showed strong agreement with the microscopic technique, confirming the reliability of the former as a diagnostic tool for *S. mansoni* detection [28].

*Schistosoma intercalatum* and *S. guineensis* have long been the subject of taxonomic debate, and are currently considered part of the *S. haematobium* species clade. As a novel aspect of this study, we evaluated the detection of these species in stool samples using the Dra1 repetitive sequence as the molecular target, which is commonly used for the diagnosis of UGS. Regarding Dra1 real-time PCR, all positive cases identified with *S. intercalatum/S. guineensis* eggs by microscopy were also Dra1 DNA positive; however, one sample tested positive for Dra1 DNA but negative for *S. intercalatum/S. guineensis* eggs by microscopy. Importantly, all six putative *S. intercalatum/S. guineensis* cases originated from Equatorial Guinea, an area endemic for this infection, providing support for their identification. Furthermore, urine samples from patients diagnosed with *S. intercalatum/S. guineensis* tested negative for *S. haematobium*, both by microscopy and real-time PCR, effectively ruling out infection with *S. haematobium* and supporting the likelihood of *S. intercalatum/S. guineensis* infection. These results show that the Dra1 repeat offers high detection sensitivity and may serve as a molecular diagnostic marker for *S. intercalatum/S. guineensis* diagnosis [36]. Nonetheless, we cannot entirely exclude the possibility of cross-amplification due to hybrid or zoonotic *Schistosoma* species, due to the lack of molecular characterisation of the eggs. However, these findings do highlight the potential utility of molecular techniques targeting Sm1-7 and Dra1 in improving schistosomiasis diagnosis, particularly in low-burden infections where conventional methods may lack sensitivity [27, 33].

When comparing real-time PCR targeting Dra1 and Sm1-7 with pan-*Schistosoma* real-time PCR, the assays targeting the species clades demonstrated higher sensitivity, detecting more cases accurately. Interestingly, the cases that tested negative with pan-*Schistosoma* real-time PCR but positive with either the Sm1-7 or Dra1 targets correspond to those with higher Ct values. This observation suggests that the amount of parasite DNA in these samples was lower, and the Sm1-7 and Dra1 assays, which target highly repetitive sequences, may offer increased sensitivity in low-intensity infections. Consequently, an effective strategy for intestinal schistosomiasis screening in non-endemic countries, when infection with *S. intercalatum/S. guineensis* and/or *S. mansoni* is suspected, is the use of a multiplex real-time PCR on stool samples incorporating both the Dra1 and Sm1-7 targets. This single assay enables the simultaneous detection of *S. intercalatum/S. guineensis* and *S. mansoni*, the main intestinal *Schistosoma* species circulating in sub-Saharan Africa and commonly found in migrants and travellers. However, because this technique requires trained personnel, dedicated equipment and a stable electricity supply, it is not suitable as a field-based diagnostic tool in rural, low-resource areas endemic for schistosomiasis, where microscopy remains the reference method due to its low cost and the minimal infrastructure required for its use. In contrast, the multiplex format provides operational advantages in high-resource settings by consolidating targets into a single assay, thus reducing cost and conserving sample material [37–39]. This approach is therefore especially relevant for the non-endemic settings, where accurate individual diagnosis is essential and where patients often present with very low parasite burdens.

This study had several limitations. First, the relatively small number of patients included in the study, which was constrained by the study design and the logistical challenges of collecting samples from each patient. Second, only a single sample per patient was available for analysis, limiting the possibility for repeat testing. Third, the inability to perform sequencing on the positive samples; egg isolation from stool followed by molecular characterisation would have allowed for definitive species confirmation. Thus, further research is needed to enhance species-level identification, particularly for *S. intercalatum* and *S. guineensis*.

## Conclusions

The results of this study highlight the growing importance of accurate diagnostic tools for schistosomiasis, and underscore the ongoing clinical and diagnostic challenges of imported schistosomiasis in high-resource settings. Despite its limitations, microscopy remains the reference standard for schistosomiasis, and continues to be a valuable diagnostic tool due to its low operational cost and feasibility of use in low-resource settings. The choice of diagnostic technique for schistosomiasis should be guided by multiple factors, including infection burden, available resources, technical expertise, and the specific epidemiological context. Our results demonstrated the limited sensitivity of the current pan-*Schistosoma* real-time PCR protocol for stool samples, with microscopy identifying a greater number of positive cases. In contrast, the real-time PCR assays targeting Sm1-7 and Dra1 showed higher sensitivity and strong concordance with the microscopic technique employed. Importantly, our findings extend the applicability of the Dra1 target beyond its well-established use for the detection of *S. haematobium* in urine, providing new evidence supporting its potential for identifying *S. intercalatum* and *S. guineensis* in stool samples. These findings suggest that a multiplex real-time PCR incorporating both Sm1-7 and Dra1 targets could serve as a practical, sensitive, and cost-effective approach for intestinal schistosomiasis screening in migrants and travellers from regions that are endemic for *S. mansoni* and *S. intercalatum/S. guineensis*.

## Supplementary Information

**Additional file 1: Figure 1S.** Egg sample visualisation under microscopy (×10 ocular, ×40 objective lens). **A** *Schistosoma haematobium* egg in a urine sediment sample,** B** *Schistosoma mansoni,* and** C** *Schistosoma intercalatum/S. guineensis* eggs in stool samples processed using Ritchie’s formalin-ether concentration technique.

## Data Availability

All data generated or analysed during this study are in the article.
