# Supplementary figures and images for: Comparison of three real-time polymerase chain reaction protocols for the diagnosis of imported schistosomiasis in a non-endemic setting
Source: Parasit Vectors. 2025 Dec 29;19:57. doi: 10.1186/s13071-025-07203-1 (PMC12859907; doi:10.1186/s13071-025-07203-1)

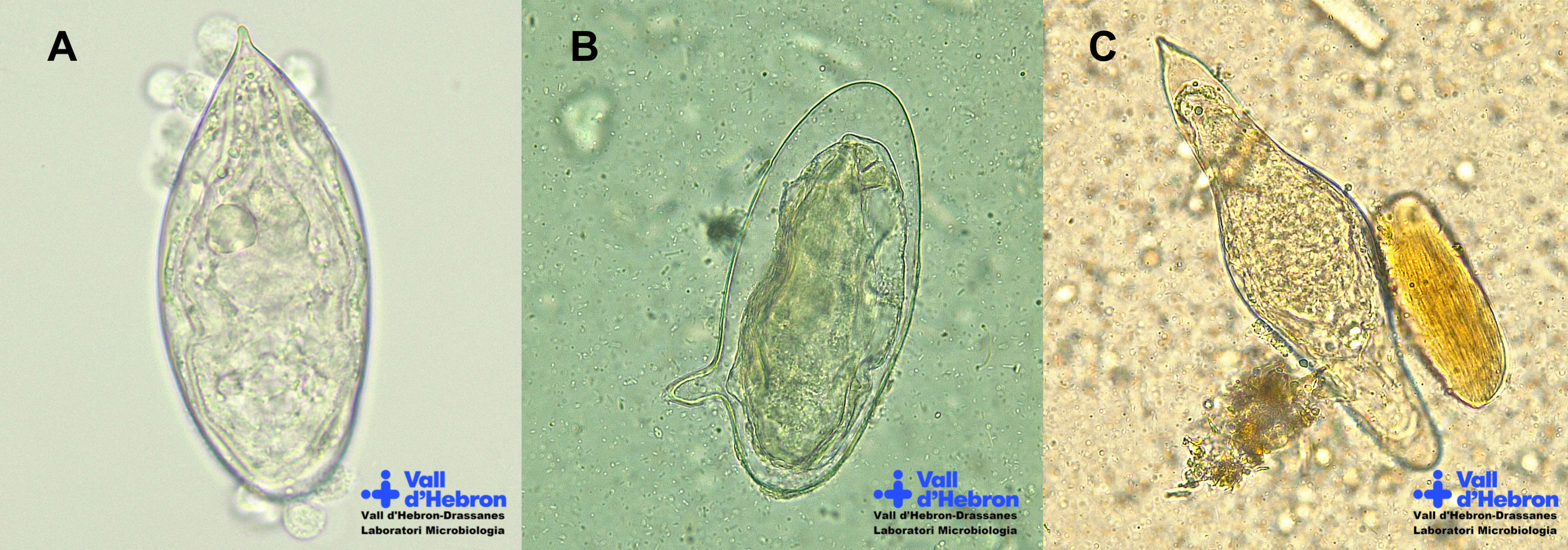

Supplement: Supplementary file 1 — Additional file 1: Figure 1S. Egg sample visualisation under microscopy (×10 ocular, ×40 objective lens). A Schistosoma haematobium egg in a urine sediment sample, B Schistosoma mansoni, and C Schistosoma intercalatum/S. guineensis eggs in stool samples processed using Ritchie’s formalin-ether concentration technique. [file 13071_2025_7203_MOESM1_ESM.tiff]
